# Unravelling the complexity of the relationship between social support sources and loneliness: A mixed-methods study with older adults

**DOI:** 10.1371/journal.pone.0316751

**Published:** 2025-01-03

**Authors:** Esteban Sánchez-Moreno, Lorena Patricia Gallardo-Peralta, Vicente Rodríguez-Rodríguez, Pablo de Gea Grela, Sonia García Aguña

**Affiliations:** 1 Department of Sociology: Methods and Theory, Research Institute for Development and Cooperation, Universidad Complutense de Madrid, Madrid, Spain; 2 Department of Social Work and Social Services, Faculty of Social Work, Universidad Complutense de Madrid, Madrid, Spain; 3 Research Group on Aging (GIE-CSIC), Consejo Superior de Investigaciones Científicas (CSIC), Madrid, Spain; Centre d’Estudis Demogràfics: Centre d’Estudis Demografics, SPAIN

## Abstract

Loneliness is an increasingly significant social and public health issue in contemporary societies. The available evidence suggests that social support is one of the key psychosocial processes for the reduction and prevention of loneliness. This study investigated the role played by sources of social support in the experience of social and emotional loneliness, identifying seven sources of support split between family (spouse/partner, children, grandchildren, siblings) and non-family (friends, neighbours). The study population comprised people aged 65 years and over living in Spain, with a partner (without cohabiting children), alone or in a nursing home. A mixed-methods approach was used, combining data from a survey involving 887 participants (quantitative phase) and data from semi-structured interviews with 30 older adults (qualitative phase). The relationship between the various sources and loneliness was analysed using structural equation modelling (SEM) for the survey data and thematic analysis for the qualitative information. The results from both phases of the study suggest different association dynamics between sources of social support and the social and emotional dimensions of loneliness. Lower levels of emotional loneliness were related to support from the following sources: spouse, children, grandchildren, siblings and friends. Lower levels of social loneliness were related to support from the following sources: spouse, grandchildren, siblings and friends. In contrast, greater levels of emotional loneliness were related to support from neighbours and greater levels of social loneliness were related to support from children. The findings of this study contribute to a better understanding of the association between social support and loneliness and suggest that interventions aimed at reducing loneliness could be more effectively targeted by considering the specific effects of support derived from different sources.

## Background

The well-known U-shaped association between age and loneliness [1] places older adults in a situation of clear risk, resulting in catastrophic consequences for this group. According to a recent report from the Joint Research Centre (JRC) of the European Commission, “both lonely and socially isolated older adults face substantial increased health risks of premature mortality (equal to smoking and obesity), developing dementia, of artery disease or stroke” [2]. Previous research has also established a clear association between loneliness and cardiovascular illnesses [3], in addition to reliably confirming risks to mental health among older adults, with loneliness linked to higher levels of psychological deterioration [4] and increased symptoms of depression [5–7]. In their systematic review, Van As et al. [8] concluded that there is remarkable evidence of both a longitudinal association between loneliness and depressive symptoms and an unfavourable course of depression associated with loneliness. In short, we are in a position to state that loneliness is a global public health problem [9]. This makes it particularly important to develop a detailed understanding of the situations, processes and factors that are associated with increased levels of loneliness among older adults, so this knowledge can be incorporated into prevention, support and intervention programmes.

Social relations are a core concept in this context [10, 11]. Loneliness can be understood as the unpleasant and disturbing experience that occurs when a person’s network of social relations is quantitatively or qualitatively deficient in some important way [12], creating a situation in which “the number of existing relationships is smaller than is considered desirable, as well as situations where the intimacy one wishes for has not been realized” [13]. Two dimensions of loneliness can be distinguished [14]. Social loneliness occurs when a person lacks a sense of social integration or community involvement, producing a failure in social connectedness. On the other hand, emotional loneliness is based on the absence of personal, intimate relationships, causing a lack of close emotional attachments. This conceptual framework establishes social relations as crucial elements in understanding the risk of experiencing loneliness. Specifically, it highlights the way that people interpret the role of those relations in their lives. In this regard, the empirical evidence has shown that the structural features of social networks (network composition and density, frequency of contact) are less significant than the quality and satisfaction offered by social contact and relations [10]. In other words, loneliness does not necessarily coincide with situations involving objective social isolation. Rather, the qualitative content of exchanges and interactions within the framework of social networks is the fundamental factor influencing the risk of experiencing loneliness. Moreover, there is empirical evidence suggesting that the relationship between social isolation and loneliness weakens over the course of a lifetime [15].

This study therefore analyses the role played by social support, one of the emergent qualitative features of social relations with the greatest potential impact on levels of loneliness among older adults [16]. We use this concept to refer to the reciprocal provision of significant resources that are an emergent feature of social relations and usually meet certain social or psychosocial needs [17, 18]. This means that social support takes effect through a wide range of life situations that pose needs linked to positive emotions such as affection, love, kindness, empathy, sympathy and esteem (emotional support), and those raising needs linked to material and financial resources, the resolution of everyday problems, and the provision of useful information or advice (instrumental support) [19, 20]. In this vein, social support consists of a series of functions performed for individuals by members of their support network. As these functions are obviously constructed within social relations, a strong connection can be expected between social support and loneliness among older adults.

Social support indeed provides a range of coping resources that are particularly important in properly managing changes in psychosocial needs linked to the ageing process, including a potentially increased risk of experiencing loneliness. It is hence no surprise that there is notable evidence of the relationship between social support and loneliness in the ageing process [21]. The specific features of social support in the case of older adults are worth highlighting. Theoretical models of social support linked to ageing emphasise the importance of changes in the composition and functioning of social networks over the life course. This is true of the convoy model specifically developed by Kahn and Antonucci [22] to understand the role of social relations in the wellbeing of older adults. According to its original formulation, individuals obtain support from a personal network made up of relatives, friends and significant others [22]. This personal network should be conceptualised from the lifespan perspective, in the sense that “the individuals are surrounded by supportive others who move with them throughout the life course” [23]. These relationships typically vary over time in terms of closeness, quality, functions and structure. The convoy metaphor is particularly useful to address one of the most important elements in the study of social support among older adults; namely, the role played by the various sources of social support and their differing impacts on wellbeing [24].

Along these lines, Carstensen [25] proposed that the ageing process involves increasing change across all domains of life: work, functionality (often linked to health problems), adjustments to social roles and positions, and changes to family and social relationships in general. According to Carstensen [25], this set of changes creates a need to focus experiences on the present moment, which often comes with a need for emotional regulation. Combined with the accumulation of losses in older people’s social networks, this process results in relatively small social networks, concentrated into social partners who are particularly important in meeting older people’s emotional needs. In other words, older adults typically “hone their social networks such that available social partners satisfy emotional needs” [26], which increases the prominence of friendships alongside spousal and family (especially parent-child) relationships. In the specific case of the relationship between social support and loneliness, the empirical evidence has emphasised the importance of marriage and spousal support owing to its negative association with different measures of loneliness [27, 28], although this relationship is influenced by the quality of the spousal relationship [29]. This means that among married older adults, the experience of marriage appears to be particularly important in understanding loneliness. Ayalon et al. [30], for example, reported that this experience includes the positive aspects of marriage (perceived social support, company, boosted esteem) but also its negative elements (including conflictive relationships or perception of low levels of spousal support). The issue here is the extent to which the marriage provides an appropriate level of intimacy and a relationship of mutual trust, which would be related to lower levels of loneliness. In addition to spouse/partner relations, various studies have noted the role played by friendships, which are particularly significant in terms of the provision of social support for older adults [31, 32], thereby contributing to reduced levels of loneliness [33, 34].

However, beyond the role played by support from spouses and friends (and, to a lesser extent, children), there is little in-depth knowledge regarding the differences in the impact on loneliness of support from different sources [35]. Specifically, we can identify two particularly significant gaps. First, as stated, the bulk of research has focused on the role of social support from spouses, friends and, to a lesser extent, children. However, there is significantly less comprehensive empirical evidence regarding other sources of support, such as grandchildren, siblings and neighbours. Additionally, our understanding of the potential differences between support from sons and daughters in terms of mitigating loneliness among older adults remains scarce. This means that there is limited available empirical evidence on the specific features of the association between sources of social support and loneliness, despite the importance of this association given that the ageing process entails significant changes to the composition and dynamic of social support networks (as noted above). Second, very few studies have incorporated the distinction between emotional and social loneliness and their relationship to sources of support [36]. As a result, we do not have a detailed analysis of the role played by the different sources of social support for each of these dimensions. Nonetheless, this knowledge would help to develop an accurate understanding of differences in processes related to the various dimensions of loneliness, information which would be highly useful in guiding social interventions and accompaniment. This study makes a contribution to closing both of these gaps by empirically distinguishing support from seven sources (spouse/partner, sons, daughters, grandchildren, siblings, friends and neighbours) and analysing their specific relationship with the social and emotional dimensions of loneliness. In addition, a design combining quantitative and qualitative techniques offers rich and detailed information regarding the relationship between social support and loneliness as well as an examination of the underlying psychosocial processes.

## Methods

### Study design and target population

We used a mixed-methods sequential explanatory design consisting of quantitative data and analysis (survey) in phase 1 (2022), followed by qualitative data collection and analysis (in-depth interviews, 2023) in phase 2. This design was chosen based on two factors. First, the study aims required a quantitative approach to allow for an integrated/joint analysis of the association between the seven sources of social support under consideration (as well as distinguishing between sons and daughters) and loneliness, offering a model that would reflect the complexity of this pattern of association for social loneliness and for emotional loneliness. Second, a qualitative phase was designed to extend the findings from the quantitative phase. Specifically, the aim was to analyse in greater depth the social, relational and psychosocial processes that would explain the patterns of association between the various sources of support and the dimensions of loneliness. Our proposal was that this triangulation of quantitative and qualitative information would facilitate an increased understanding of the relationships between social relations and loneliness. In this regard, most available empirical evidence examines the relationship between social support and loneliness from a quantitative perspective [21, 37]. It is hence reasonable to proceed using designs that make it possible to frame quantitative findings within a broader understanding of the experience of loneliness, as described by individuals in connection with their social relations. This makes it possible to identify and provide a detailed description of the coincidences and convergences that can arise between the findings from both study phases. In this context, we anticipated that the combination of quantitative and qualitative studies would provide a rich and full exploration of the connections between social support from different sources and social/emotional loneliness.

The study’s target population was made up of individuals aged 65 years and above and living in Spain (country of residence) with a partner (no other people living at home), living alone, or having been resident in a nursing home for more than six months. Older adults living with a partner and someone else (e.g., son/daughter) in their household were excluded. The decision to use living arrangements as an inclusion criterion was based on the available empirical evidence regarding the remarkable impact of the three situations taken into account in this study on levels of loneliness experienced by older adults when compared with other living arrangements [38, 39].

### Quantitative phase

#### Survey data collection

The quantitative data were collected using a survey administered by trained individuals between 16 May and 7 July 2022. A total of 887 Spanish residents took part in the study. Given the study aims, it was very important to access the general population living in any of the arrangements under consideration. In this vein, participants were a nationally representative sample for the target population, recruited through the services of the “Political and Social Analysis” research group of the University of Santiago de Compostela (Spain) were engaged for this purpose. This group has broad experience in conducting research surveys (those designed for academic purposes). Initial contact was made by telephone in all cases. The survey was applied by telephone call to the community-dwelling participants, except in cases where a face-to-face meeting was requested. For nursing home residents, the surveys were applied in face-to-face meetings. After the initial contact was made with potential participants and their informed consent had been obtained, the surveys were applied under the conditions chosen by those who agreed to participate. Nursing home residents were interviewed at a space in the nursing home that guaranteed the confidentiality of their interview. After eliminating participants who failed to provide information for any of the study variables, the final sample comprised 863 people. The minimum sample size was 108, calculated with G*Power [40] under the assumptions of a two-tailed test, α = .05; β = .01 (95% power); medium effect size (0.5) and 60 predictors.

#### Survey measurement

The survey included a block of sociodemographic questions that incorporated the variables of sex, age, having children, education (incomplete primary education, completed primary education, secondary education and university education), living arrangements (living with partner only, living alone and living in a nursing home) and limitations on activities of daily living due to health problems. The latter variable was evaluated using the Global Activity Limitation Indicator (GALI) [41].

A second block of questions incorporated measures of loneliness and social support. Loneliness was assessed using the six-item version of the de Jong Gierveld loneliness scale (DJGLS) [42]. The DJGLS was validated in Spain by Ayala et al. [43] for a sample of older adults. It is a short and commonly used instrument in loneliness research that produces an overall score and two subscores for social and emotional loneliness. This multidimensional approach makes this instrument the most appropriate one to achieve the aims of this research. Three items are used to calculate the emotional loneliness score (e.g., “I miss having people around me”) and another three are used for social loneliness (e.g., “There are plenty of people I can rely on when I have problems”). Each question has three response categories (“yes”, “more or less” and “no”). Items are scored on a scale from 0 to 2, although they are subsequently recodified as dichotomous (0 or 1). In this recodification, following the author’s guidance [43], the middle category (“more or less”) indicates loneliness, together with the category corresponding to the direction in which the question is formulated (e.g., “yes” for the item “I miss having people around me” and “no” for the item “There are plenty of people I can rely on when I have problems”). Higher scores on the scale indicate stronger feelings of loneliness (range from 0 to 6 for the total score, and from 0 to 3 for the emotional and social dimensions). The total scores for both subscales (emotional and social loneliness) were used as outcome variables for the analysis. In addition, the DJGLS allows the use of a cut-off point of 2 or higher to classify participants as experiencing loneliness [44, 45]. In the present study, this cut-off point was used exclusively to provide an estimate of the prevalence of loneliness in the sample of older adults (see Table 1). For the remaining analyses, the scores were used as continuous variables.

**Table 1:** Joint display of integrated data collection.

Topic	Quantitative findings / measurement	Qualitative material (topic in the interview)^1^	Rationale and/or theoretical background
1. Experiencing loneliness: emotional and social	De Jong Gierveld Loneliness Scale (DJGLS)	**Open questions**: Would you say you are lonely? Do you feel that at this stage of life it is more likely to feel lonely?	Explore the multifaceted nature of loneliness by integrating quantitative measures and subjective accounts
2.1. Pattern of relationship between social support and loneliness	Complex relationship: different association patterns for emotional and social loneliness (multivariate analysis, SEM)	**Open questions**: In what situations do you feel most lonely? In what situations do you feel most accompanied?	Understand the importance of social relationships in the experience of loneliness among older adults Create a social network map based on spontaneous nominations of significant people
2.2. Contribution of different sources of support		**Open questions**: On an ordinary day, who do you relate with? Of those people, who is more important for you? Do you meet these people often, or would you like to meet them frequently? **Open question**: If you need something, who do you turn to? In what situations do you most value receiving help or being able to turn to someone? Emphasis on the spontaneous occurrence of different sources within the discourse	**Background**: Convoy Theory and Emotional Selectivity Theory **Rationale**: Elicit reasoning about how different relevant people orient themselves towards participants, given that they are older adults Produce qualitative data where participants establish patterns of relationship with their different support sources, within the context of their daily lives
*Partner*	Key persons for low scores on social and emotional aspects of loneliness		
*Friendship*			
*Siblings*			
*Sons and daughters*	Statistically significant positive association with loneliness		
*Grandchildren*	Statistically significant negative association with loneliness (especially with social aspect)		
*Neighbours*	Statistically significant positive association with loneliness		

^1^These questions are a carefully selected subset from the interview script, chosen for their usefulness in illustrating the link between the quantitative and qualitative phases of data collection.

Functional social support was assessed using the Perceived Social Support Questionnaire (PSSQ) [46]. This scale was originally developed to measure social support among the Spanish-resident population. This instrument provides separate scores for the different sources of social support, offering a notable opportunity to include the specific sources of interest for each study. For each source, two items are used to measure emotional support (e.g., “Could you freely express and share your emotions with this person?”), two for instrumental support (e.g., “If you were sick or needed to be taken to the doctor, would this person be of any help?”), and two for advisory support or guidance (e.g., “Would this person be of any help if you had to make an important decision?”). Scores for the items corresponding to each source and representing the different types of support (emotional, instrumental, advisory) produce a composite functional support score for each source. The responding scale for each item ranges from 0 to 5, and the sum for each source (higher scores representing higher levels of social support provision) was standardised to a 0–10 range. In our case, the scale was adapted to measure support from the seven sources under consideration. As a result, in this study the PSSQ comprised 42 items with questions on the source and level of perceived social support from spouse/partner, sons, daughters, grandchildren, siblings, friends and neighbours. The PSSQ is a widely used instrument in Spanish-speaking populations, having been validated in various contexts (residential, general and hospital) [47, 48].

#### Quantitative data analysis

**Fig 1:**
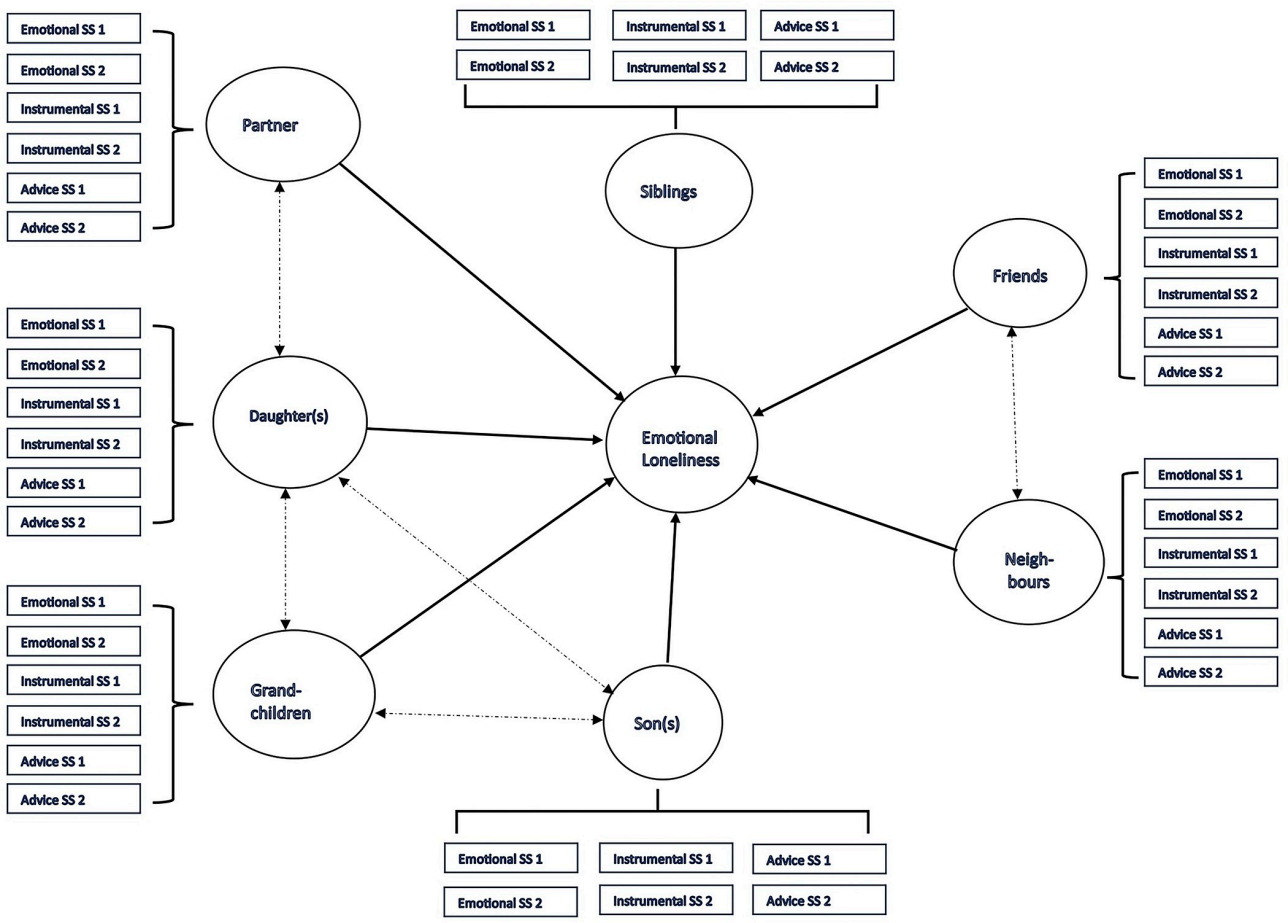
Baseline model contrasted with empirical data via structural equation modelling.

First, descriptive statistics were used to present the respondents’ demographic characteristics. Second, prevalence of risk of loneliness was calculated using the above-mentioned cut-off score and reported as percentages of cases in each subgroup. Chi-square tests were used to determine demographic characteristics associated with risk of loneliness. Both steps offer useful contextual information. Third, the central analysis for this study was conducted: contrasting a multivariate model of the association between loneliness and social support. Structural equation modelling (SEM) was used for this purpose, including both the measurement model (the aforementioned loneliness and social support surveys) and the structural model (association between latent variables). This analysis used the total scores obtained for the relevant variables (emotional/social loneliness and social support from spouse/partner, sons, daughters, grandchildren, siblings, friends and neighbours). Fig 1 summarises the baseline model that was contrasted with the data, including both the measurement model and the structural model. This model was progressively adjusted to the data, using the modification indices. Each decision to modify the baseline model was theoretically informed.

The significance level was set to p < .05 for all analyses. The fit of the SEM models was evaluated using a combination of relative fit indices and noncentrality-based indices. From the first group, Bollen’s Incremental Fit Index (IFI), the Tucker-Lewis Index (TLI), and the Bentler-Bonett Norme Fit Index (NFI) were used. From the second group of indices, the Root Mean Square Error of Approximation (RMSEA) and Bentler’s Comparative Fit Index (CFI) were used. For IFI, TLI, NFI and CFI, values exceeding or approaching .95 indicate a good fit of the model to the data [49]. More specifically, cut-off values of .99, .95, .92 and .90 are established as respectively corresponding to models achieving excellent, close, fair and poor fit. For RMSEA, values below .05 indicate a good fit, while values of .08 or lower suggest an acceptable fit [50]. It is also worth bearing in mind that large model size tends to be penalised in the calculation of indicators, whose values may deteriorate in models with a high number of parameters (observed variables) [51]. All statistical analyses were performed using IBM-SPSS (V29) and IBM-AMOS (V29).

### Qualitative phase

#### In-depth interview procedures

Semi-structured interviews were used to collect information in the qualitative phase, with 20 women and 10 men interviewed of ages ranging from 65 to 99 years (M = 76.8), of whom 13 were living alone (one-person households), 11 were only with a partner (couple without children living together) and 6 were in a nursing home. In this way, it was possible to include a sufficient number of participants, as defined by the inclusion criteria, particularly the criterion regarding living arrangements. The interviews were conducted between 12 September and 20 December 2022. Two participant identification and selection strategies were used. First, for the selection of community-dwelling older adults, a similar approach to that used for the quantitative phase sample was applied, involving engaging the services of the Foundation for Advanced Social Research. This not-for-profit foundation is dedicated to supporting research work and offers extensive experience in the extraction of samples for qualitative studies. It works with research groups in university settings and hence has detailed knowledge of academic research practices and standards. Second, in the case of nursing home residents, the research team directly contacted the nursing homes and provided information on the aims and ethical aspects of the study. Nursing home staff identified potential participants and established initial contact, after which arrangements were made to visit the nursing home and conduct the interview.

The inclusion criteria used for the study (see “Study design and target population” subsection) formed the starting point for participant selection. The identification of potential participants also incorporated an age-based criterion to ensure that the sample included a range of ages and avoided focusing on a narrow range (such as 65 to 75 year olds). It was also established that a significant number of participants had to be male. In this regard, the living arrangements included in the study implied a risk that the sample might be almost exclusively female, given their greater life expectancy and the feminine structure of the population aged over 65 years in Spain. The initial contact was used to confirm that the potential interviewee was part of the target population. Following confirmation that an individual was prepared to participate, a member of the research team then attended the interview, which was arranged at university offices, the interviewee’s home or another location meeting the requirements of being a comfortable and appropriate space for an interview. In the case of nursing home residents, a social worker at the nursing home provided a space that ensured the interview would be private and confidential. The opening minutes of the interview were used to reconfirm that the person fit the study inclusion criteria in all cases.

The interviews were conducted in Spanish, took between 40 and 75 minutes and involved the application of a semi-structured script including questions related to the study aims, such as: who do you have relations with in your daily life? Who is the most important? If you have any needs, who do you turn to? How valuable is their support? What kind of support is most important for you? The main focus was the experience of loneliness in old age, however, with questions including: do you feel alone? In which situations do you feel most lonely? Do you think there is a higher chance of feeling alone during this stage of life? Which circumstances can affect feelings of loneliness in old age? The script was designed to be flexible and relatively unstructured, making it possible to incorporate significant emerging topics into the dialogue. Table 1 presents a joint display illustrating the integration of quantitative and qualitative data collection instruments. The final column outlines the rationale for combining these methods. The qualitative questions included in the table are a subset of those included in the full script (see S1 File).

#### Qualitative material analysis

All interviews were audio-recorded, transcribed and imported in Spanish into Atlas.ti (V.23) for analysis. First, the interviewees’ statements were used to identify the most emotionally close social networks. The in-depth interview script included an open-ended question designed to elicit participants’ perceptions of their most significant social relationships. This approach allowed for a more spontaneous and less structured identification of key social ties than a closed-ended questionnaire. The resulting transcripts were used to construct a database where the columns represented the 30 interviewees and the rows listed all the significant individuals mentioned. This information was processed using UciNet 6 software [52] to graphically represent the interviewees’ significant social relations. Second, a thematic analysis of the thirty interviews was conducted [53, 54]. Our approach to thematic analysis was influenced by the sequential explanatory design of our research. As noted in the “Study design and target population” subsection, the qualitative data collection phase was informed by the quantitative data collected in the first phase of the research. Furthermore, our thematic analysis aimed to deepen and expand our understanding of the model of association between social support and loneliness identified in the quantitative analysis. Finally and notably, our research design was based on a theoretical elaboration of the available empirical evidence, as analysed in the first section of this paper. As a result, our approach to coding and identifying themes was primarily deductive [53], driven by our analytic and theoretical focus on the complex relation between sources of social support and the dimensions of loneliness. The thematic analysis was completed in the following steps. The analysis started with familiarisation with the data (reading and re-reading). As part of this first step, the second author produced the initial codes in Atlas.ti linked to the research questions, focusing on the qualitative aspects (degree of emotional closeness, satisfaction with ties) and functional aspects (type of social support received and reciprocity) of the social relations and the experience of loneliness (dimensions and feelings of loneliness). Reflective notes were used throughout the process [55], a very useful tool for organizing information into meaningful groups. This initial set of codes was examined and refined by the first two authors in a close review of transcripts and interview summaries. The second step addressed the construction of themes [55]. The results of the quantitative analysis theoretically and analytically oriented both the search for themes and the relationship between them. The research team worked as a group and collaboratively to organize codes by giving a common meaning to those groups of codes that collapsed into emerging themes. These themes were classified into main themes and sub-themes, generating a provisional thematic map. The research team then identified and developed broader patterns and combinations of themes until they reached a consensus regarding the key themes of the interviews linked to the features of social support (by source) and their association with feelings of loneliness. Subsequently, the second author reviewed this collection of potential themes, contrasting it with the qualitative material; that is, with the excerpts and codes identified as an expression and part of each theme. This work produced the final thematic map, which the team subsequently analysed in an integrated manner together with the results of the quantitative phase.

#### Ethical considerations

All study procedures were approved by the Universidad Complutense de Madrid ethical committee (report reference CE_20220217–14_SOC). Emphasis was placed on protecting participant confidentiality in both phases of the study (quantitative and qualitative). Participants were individually informed about the nature of the study and its risks and benefits. Notable benefits included the contribution to generating knowledge in a field of study (loneliness) that is particularly significant for society in general and for older adults in particular. In terms of risks, participants were warned that the interview included themes of family and social relations, which might entail discussing unpleasant or sad situations. Each participant had the opportunity to read their interview transcript prior to codification and analysis. Only one person requested to do so. In the quantitative phase, verbal consent was obtained. The record of this consent constituted the first item of the survey, which could not be continued unless the corresponding box was checked. Written consent was obtained in the qualitative phase, and all signed documents have been archived and are currently in the custody of the lead researcher. Participants in the quantitative phase were not paid for participating in the study, but qualitative-phase participants did receive payment.

## Results

The results are presented as follows. First, participants’ characteristics are summarized and bivariate analyses provided. In addition, a visual representation is presented that shows participants’ support networks. Second, the quantitative and qualitative findings are jointly presented in view of their high level of consistency and complementarity. This presentation style adequately reflects the decision to integrate quantitative and qualitative data through a narrative approach [56] on a theme-by-theme basis. This made it possible to offer a detailed view of the complex association between social support and loneliness in our study results.

### Participant characteristics, prevalence of (risk of) loneliness, descriptive and bivariate analysis

The total sample included 539 women (62.1%) and 700 respondents had children (80.6%). The participants’ mean age was 78.5 (SD = 8.7). In terms of education, 386 (44.8%) respondents had completed primary education, while 198 respondents (23.7%) had not; 130 participants (15%) had completed secondary education, and 145 (16.6%) had obtained university qualifications. As regards living arrangements, 330 respondents (37.4%) were living alone, 369 (41.8%) were living with their partner only (no cohabiting children) and 164 (20.7%) were living in a nursing home. Finally, 466 respondents (54.1%) were not restricted by health problems, 298 (34.5%) were restricted to a moderate degree, and 95 (10.9%) faced serious restrictions.

The prevalence of loneliness (using cut-off point 2 on the DJGLS) was 43.3% (n = 374). Table 2 shows the differences in prevalence of loneliness based on sociodemographic characteristics. With the sole exception of sex, significant differences were found for all variables, with situations involving a risk of loneliness particularly prominent among people who did not have children (58.9%), were seriously restricted by health problems (69.5%), had not completed primary education (59.1%) or were living in a nursing home (68.3%). A significant, albeit low-scale, correlation was also found between age and loneliness (r = -.10; p < .01).

**Table 2:** Bivariate analysis for social and demographic variables and loneliness.

Variable	Loneliness (risk, cut-off point = 2)	χ^2^; *p*
***Sex***		
Women	44.2%	χ^2^ = .392 *p* = .53
Men	42.0%	
***Children***		
Have children	39.7%	χ^2^ = 19.810; *p <* .001
Do not have children	58.9%	
***Limitation on ADL***		
Not limited	33.9%	χ^2^ = 47.696; *p <* .001
Limited	49.3%	
Strongly limited	69.5%	
***Education***		
Incomplete primary education	59.1%	χ^2^ = 32.975; *p <* .001
Complete primary education	43.0%	
Secondary	34.6%	
University	31.0%	
***Living arrangement***		
Living alone	48.2%	χ^2^ = 80.495 *p <* .001
Living with partner only	27.9%	
Nursing home	68.3%	

**Fig 2:**
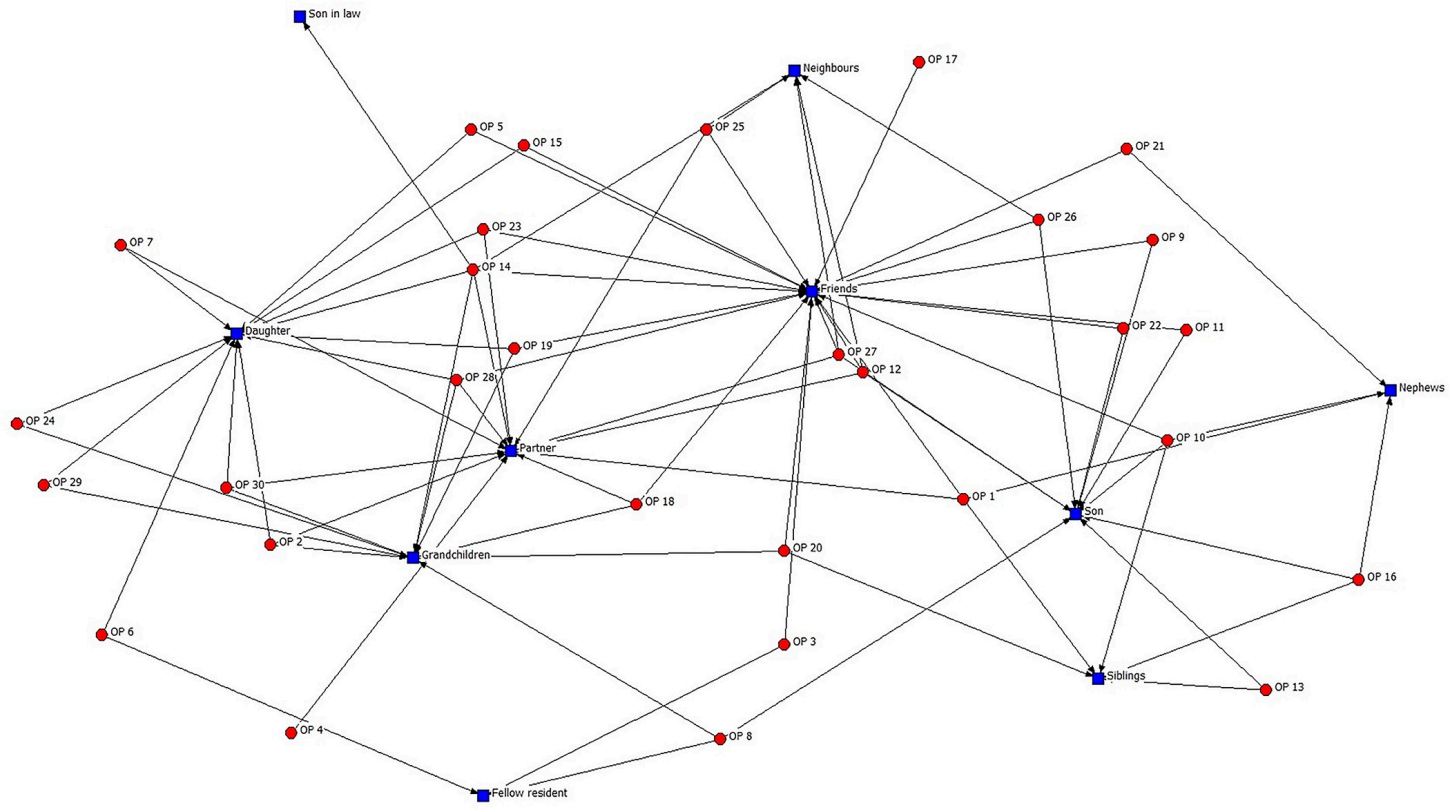
Social network members identified as significant by older adults in semi-structured qualitative interviews. (OP: older person; n = 30).

The information collected through qualitative interviews facilitated an initial examination of the relative significance of sources of social support in participants’ lives. The analysis of significant social relations using UciNet 6 (see Fig 2) identifies family relationships as notably prominent. These were spontaneous responses to a generic question concerning the most significant social relations, included in the interview script. In order of importance, we identified partner (described as a significant source on 12 occasions), daughter (12), son (10), grandchildren (10), siblings (5), nephews (4) and son-in-law (1). However, it is worth noting the great importance of friendships, mentioned by 21 interviewees as a significant source. Neighbours (5) and fellow nursing home residents (3) were identified much less frequently.

### Multivariate and qualitative analysis: A general model of the relationship between social support, its sources and loneliness

The results described so far offer an initial overview of the relationship between social support from various sources and loneliness. However, bivariate analyses do not offer an understanding of the potential complexity of the relationship pattern between these processes. In addition to the direct links between two variables taken separately (in this case, loneliness and social support from various sources), it is advisable to analyse the direct and indirect multivariate relationships of all the variables considered: in this case, relationships between the sources of social support, on one hand, and with loneliness and its dimensions, on the other. SEM analyses were carried out for this purpose, and their results were triangulated with the qualitative information obtained during the in-depth interviews, as explained in the section dedicated to describing the analytical strategy. The results are set out below.

**Fig 3:**
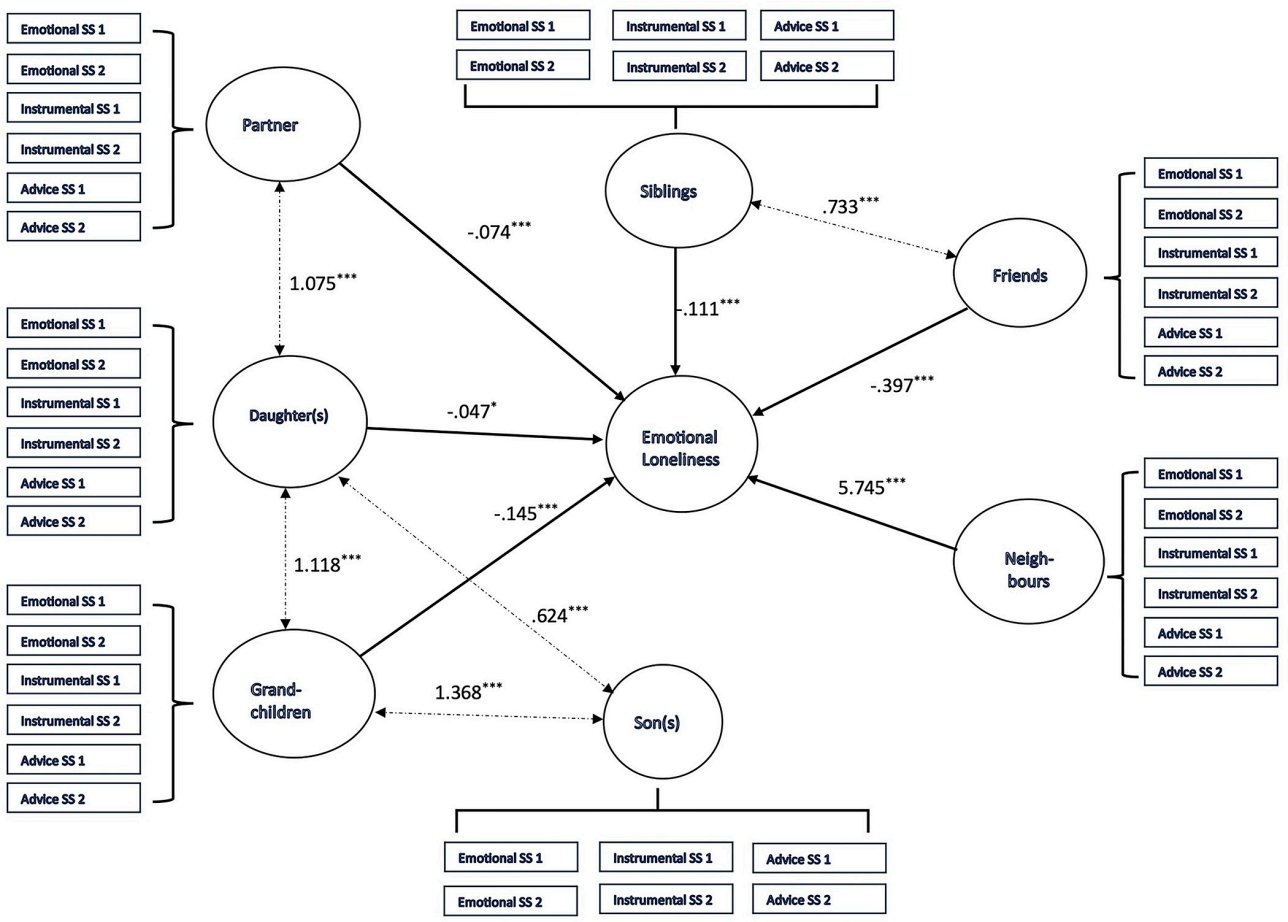
Structural equation model specifying the relationship between social support from family and non-family sources and emotional loneliness [***p < .001; ***p < .01; ***p < .01; IFI = .931; TLI = .926; NFI = .920; RMSEA = .083; CFI = .931].

**Fig 4:**
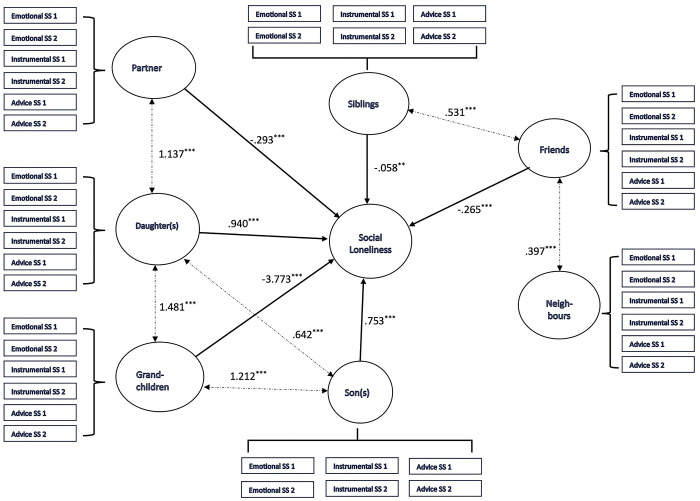
Structural equation model specifying the relationship between social support from family and non-family sources and social loneliness [***p < .001; ***p < .01; ***p < .01; IFI = .934; TLI = .929; NFI = .924; RMSEA = .081; CFI = .934].

The SEM analysis results are presented in Fig 3 (emotional loneliness) and Fig 4 (social loneliness). The measurement model and structural model are included in both cases. However, for ease of reading, only the coefficients corresponding to the structural model are included. The complete results, corresponding to the measurement model and the structural model, are available in S1 and S2 Tables. Both models generated fair indicators of fit to the data: the predictive model for emotional loneliness scores (Fig 3) (IFI = .931; TLI = .926; NFI = .920; RMSEA = .083; CFI = .931), and the model for social loneliness (Fig 4) (IFI = .934; TLI = .929; NFI = .924; RMSEA = .081; CFI = .934). The results suggest remarkable coincidences in the relationship pattern between social support from various sources, on one hand, and social loneliness and emotional loneliness, on the other. However, there are important nuances showing that the significance of the different sources of support varies for each dimension of loneliness (emotional and social), as discussed in the following section.

**Fig 5:**
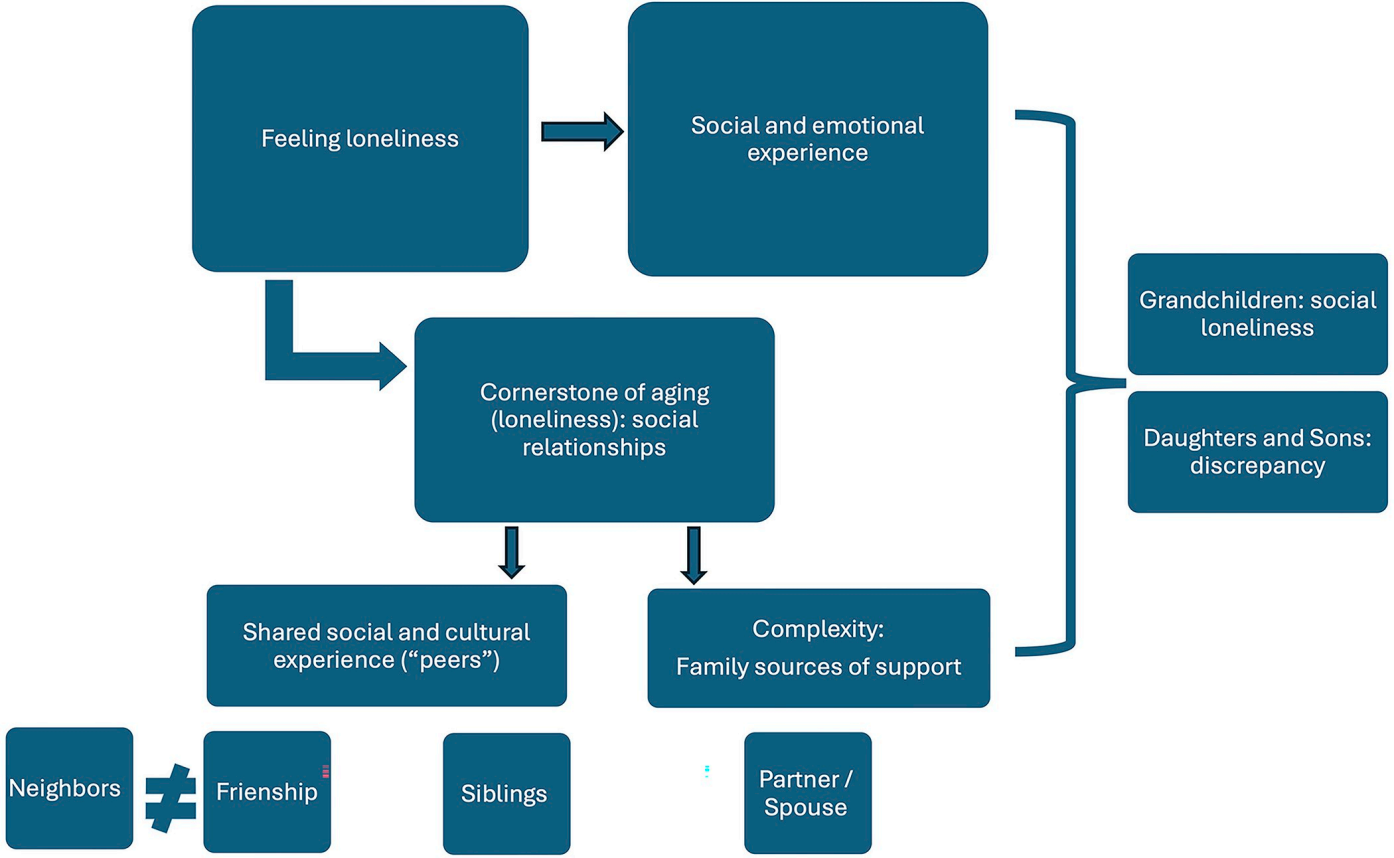
Themes arising from qualitative data on social support sources and loneliness.

Fig 5 presents the results of the thematic analysis of the qualitative data. This figure incorporates the themes that articulate the qualitative material and includes the pattern of relationships between the different themes, configuring a thematic map that summarizes the complexity of the relationship between social support from different sources and loneliness. Although a detailed analysis integrating the quantitative and qualitative data will be provided in the following pages, it is worth noting at this point the different roles that various sources (family and non-family) play in the participants’ experiences of loneliness, as described in their own discourses. As we will see, the thematic analysis summarized in Fig 5 is particularly important in expanding our understanding of the association patterns identified in the analysis of the quantitative data.

#### Social support from family and loneliness

In Figs 3 and 4, support from spouse/partner stands out as being related to lower loneliness scores. However, it is striking that the regression coefficients show its significance to be greater in the case of social loneliness (β = -.293; p < .001) than for emotional loneliness (β = -.074; p < .001). The analysis of qualitative material is aligned with these results. In all the interviews with participants living with their partner (without cohabiting children in all cases), the spouse emerged as a significant source of day-to-day support, helping participants to cope with the problems inherent to old age and acting as an emotional refuge to avoid loneliness. It is worth noting that in practice, spousal support has a combined impact in terms of reducing both social and emotional loneliness:

She really is for me [referring to her being a supporting person], she’s always looking after me, anything, she’s doing my drops, she’s reminded me twice today (…) And then at four this morning I was trying not to wake her up, she noticed me getting up and woke up because I was going to the bathroom to do my drops and there she is behind me, she’s woken up, so you can see how much my wife supports me (…) She looks after me like a boy, like a child, I mean to say she’s supporting me 100% and she’s always with me. (OP1, male, living with partner, 72 years)

Well, my husband [referring to closest support network], I don’t see my children every day (…). With my husband, we’re really close, he’s had an operation too and we’ve both got our ailments but right now we support and help each other (…) Like I’m telling you, as I wasn’t well, my husband even put my socks on. (OP12, female, living with partner, 76 years)‬‬‬‬‬

In the marriage, we both try not to bother others if we can do it ourselves. So if it’s been necessary to go to the doctor, we go, if we have to go by metro or take a taxi we go to the doctor, I mean to a consultant or to the clinic, we always go on our own, there’s no need because we can look after ourselves fine for the time being, thank God (…) But there are day-to-day things that we don’t bother about. (OP18, male, living with partner, 81 years)

The association pattern between social support and loneliness is significantly more complex in the case of sons and daughters. Support from the latter was related to lower scores for emotional loneliness (β = -.047; p < .05), although there was a smaller association in comparison with other sources (spouse, siblings, grandchildren and neighbours). It is worth emphasising that there is no significant association between emotional loneliness and support from sons. But there was a positive association between social loneliness and both sons (β = .753; p < .001) and daughters (β = .940; p < .001), such that higher scores for the support measure were associated with higher scores for social loneliness. The material collected in the interviews offered highly useful context to analyse the complexity of the relationship between emotional and social loneliness, on one hand, and social support from sons and daughters, on the other. In fact, the interviews revealed a clear distinction between the two. Specifically, the interviewees described daughters as a key source of emotional support in their lives, more easily creating a relationship of attachment based on communication, intimacy and mutual care. However, this relationship may be affected by difficulties maintaining frequent social contact:

My daughter, my daughter is by far the most important person or thing in my life (…) we talk a lot in our relationship, yes. We’re lucky to live fairly close by but she’s really busy with work too (…) so she’s quite pushed for time, and what we do is talk on the phone for the hour it takes her to go to work, and that’s an hour every day, whether or not we see each other, so in that hour you can imagine, we talk about everything that has happened to us during the day, everything we’ve seen, everything we’ve been told, everything under the sun, we talk about ourselves and everything around us, so there really is lots of communication in our relationship. Regardless of when we need to be there for everything (…) it’s a bond we have and I hope that all mothers and daughters are like that. (OP5, female, living alone, 67 years)

Sons and daughters were frequently compared in the interviews. It was a significant theme for the participants:

Yes, whenever I’ve needed it, because I’ve already needed them to take care of me, it’s been my daughter who’s been there unconditionally, and two friends of mine who’ve even brought me food, they’ve gone with me to the doctor, and all that. If I’ve occasionally asked my son to do something or other, he hasn’t refused, but he hasn’t volunteered or checked if I’ve needed help. (OP4, female, living with partner, 73 years)

More specifically, the interviews indicated a tendency for sons to specialise in providing instrumental support, compatible with an emotional distancing that helps provide context for the data in Figs 1 and 2. In this regard, we found descriptions of support from sons as being strictly instrumental and task-focused, with far less emphasis on emotions:

My son, I can’t do anything without him, I don’t know anything. He’s the one who goes to the bank, the doctor, he does everything, he’s everything to me; I’m nothing, nothing without him. (OP10, female, living alone, 91 years)

My son has a more independent nature. So as he’s got older I think that he loves me, because he shows me, but he doesn’t need me. He knows I’m there even though he doesn’t need me. So the time he can give me or find out how I’m doing or whatever, he might prefer to chat with a friend, who thinks more like he does, or maybe he has more of a laugh with them than me, I don’t know. Because he does love me, he’s shown me, but I don’t think he feels the need for the closeness that my daughter wants with me. (OP5, female, living alone, 67 years)

In any event, it is worth noting that social support from sons and daughters significantly correlates with social support from grandchildren. This is significant: this support was one of the most important sources both in the SEM analyses and in the analysis of the in-depth interviews. In fact, perceived support from grandchildren is related to lower scores for emotional loneliness (β = -.145; p < .001), but the intensity of the association is particularly significant in the case of social loneliness, owing to its magnitude (β = -3.773; p < .001). This latter case represents the most intense association in the model set out in Fig 3, showing the importance of support from grandchildren in the social loneliness experienced by older adults. The analysis of the in-depth interviews contributes to describing this process, which is largely modulated by the age of the grandchildren. The emotional closeness of the relationship is the most significant element when the grandchildren are younger, but interaction with grandchildren generally creates specific wellbeing that is largely related to the maintenance of significant social relations:

(…) my grandchildren are indispensable, it really is a totally different kind of love, now I’m the grandfather, now they adore you. One’s eight, another is six. I give them four years, probably they won’t come and see me once they’re 12, but now they come to grandad’s house and they have a great time. (OP9, male, living alone, 68 years)

My grandchildren have gone on holidays, one of them because another is studying in the US on a grant, we’re not rich. And we call each other, I call them, they call me, they don’t come and see me as much, I’m always missing them like always. Although they’re women, one is 19 and the other 17, they’re doing really well with their studies, they get great marks and that keeps me happier too, it keeps me going. (OP8, male, nursing home, 78 years)

Family sources of support can be observed to be basic elements in understanding processes related to loneliness. As we have seen, the interviewees tend to locate their social ties involving emotional closeness and social interaction within the family setting, with widespread recognition that the family is a fundamental source of support. This is the context for the association between support from siblings and emotional loneliness (β = -.111; p < .001) and social loneliness (β = -.058; p < .01). Both cases involve negative regression coefficients. This is reinforced by the correlation with support from friends (a particularly important source of support for older adults, as discussed in the “Non-family sources of support: friends and neighbours” subsection). The importance of sibling support in reducing loneliness and the functional equivalence of support from siblings and friends are clearly described in the qualitative context:

I think so, having the sense of family with friends, siblings, nephews, who you can call at any time and say “hey, this has happened, it’s just that this has happened to me” and they say “fine, don’t worry, I’m coming”. That’s essential for me. You feel a bit sheltered, and if you need anything you know they’ll come. They might not come as quickly as you want, but they’ll come. (OP18, male, living with partner, 81 years).

My siblings, I’ve got siblings I’m very close to (…) we don’t call each other much, but I know I’ve got their love and friendship. My siblings have been really crucial for me (…). (OP26, female, living alone, 78 years)

#### Non-family sources of support: Friends and neighbours

The results of our study show a significant negative association between support from friends, on one hand, and the emotional (β = -.397; p < .001) and social (β = -.265; p < .001) dimensions of loneliness, on the other. This association is more significant in the case of emotional loneliness. Friendships are a key category in the in-depth interviews. In the qualitative material, the support generated in friendships is highly important in emotional terms, since it is linked to the existence of close bonds of trust:

I don’t know, at this time of life you can get low for any reason, I don’t know, you can have slightly depressed moments (…) and that feeling of loneliness, which I haven’t had, that’s bad. It’s one thing for me to be alone because I want to, which I normally look for (…) I value the emotional support more than money, and more than anything knowing that you have someone there who’s bothered about you, I don’t know, that’s something that I do value. Because I’ve done it, with friends, and knowing that they’re there, it’s what I was telling you, even if you don’t see them, even then… but knowing they’re there, that really gives you strength. (OP9, male, living alone, 68 years)

A particularly high number of interviewees (20 people, see Fig 2) state that friends are networks for emotional support, available on a day-to-day basis. This implies two interrelated elements that stand out in the analysis of the interviews. First, friendships create interactions with positive effects for both emotional loneliness and social loneliness. Second, the association very frequently takes the form of a day-to-day relationship:

I see my friends almost every day. We’re a group of friends who all live here in the same street, all of us are older now, and we meet up for coffee, then we go shopping. We do it every day, with or without aches and pains, some not so good, others well, but we get out (…) we tell each other our news and then we all go home, just that bit of time in the morning, we don’t go out later in the afternoon. (OP12, female, living with partner, 76 years)

The huge importance of the intimacy and trust that characterises the friendships described in the interviews means that when necessary, support from friends is functionally equivalent to the support received from certain family relationships:

My sister hardly gives me any support (…) but right now I get lots more support from my friends and my nursing home companion than the others, than my family, it’s as clear as that. I haven’t been a very family-focused person either, I’ve always had a bit of a rootless life. (OP3, male, nursing home, 67 years)

In our study, support from friends is the only non-family support with a significant role in reducing loneliness. In this regard, a significant association was not found between support from neighbours and social loneliness (Fig 4), and there was a positive association in the case of emotional loneliness (Fig 3), with higher scores for support related to higher scores for loneliness (β = 5.745; p < .001). Although these findings will be examined in the discussion section, it is worth noting that in the discourses collected in the in-depth interviews, relations with neighbours only emerge as a significant factor in reducing loneliness when they converge into friendship with those living in the same neighbourhood. This involves spending time together, supporting each other and developing a space for emotional cooperation and closeness, which forms the basis for constructing patterns of frequent social interaction:

Well, there are a few neighbours, a young woman who lives opposite my apartment, who always offers me help. I don’t have many friends here in Madrid, but I do have two, three, four or five. We have a group too, and I think if someone needs any help they can get it there. Meanwhile we’re always in touch, mostly through the chat group, through WhatsApp (…) (OP14, female, living with partner, 81 years)

I’ve got a friend who’s 86, but I don’t know. She lives on this street, just at the end, on the same side and everything, and I get on so well with her, I can talk about more with her than with others my own age, you know? I don’t know, because I can talk about everything, we go out for lunch, sometimes we go to the cinema (…) I’m just like a young kid when I’m out with her. I don’t know, she’s got a very young spirit, she lived in France for a long time and I don’t know, she gets everything, we understand each other very well (OP16, female, living alone, 65 years)

## Discussion

The results show the complexity of the association between social support and loneliness. One key feature of this complexity is the source of functional support. Some of our results are in line with the literature. Support from the family network is of great help in understanding the dynamic of loneliness among the study participants. In both the quantitative and qualitative results, the importance of the spouse is aligned with the available empirical evidence, comprising a significant source of support to reduce loneliness [30, 36], and particularly social loneliness [57]. It is particularly striking that the pattern of the association between loneliness and support from grandchildren coincides with that described for spouses: there is a negative association with the emotional loneliness measure, but the magnitude of the association is significantly greater with the social loneliness measure. In Fig 4, in fact, the highest regression coefficient measures the association between support from grandchildren and social loneliness. In the qualitative material, as stated in the previous section, grandchildren represent a particularly significant source of company and social relations for older adults. There is scarce empirical evidence specifically concerning the impact of support from grandchildren. However, our results are consistent with previous studies reporting that the relationship between grandparents and grandchildren generates significant social interactions in family contexts (gatherings, visits, celebrations) and non-family situations (particularly school-related) [58, 59]. These social relations are based on affective elements, explaining their association with emotional loneliness [60], but above all they generate long-term access to a range of meaningful social relations, characterised by frequent interaction and capable of including older adults in an engaging social network [61].

Support from sons did not show a significant association with emotional loneliness in our results, while it was associated with higher scores for the social loneliness measure. Support from daughters was also related with higher levels of social loneliness, although it was associated with lower scores for the emotional loneliness measure. The distinction between sons and daughters as sources of support is one of the key contributions of this study. In fact, there is no existing literature that separately analyses their relationships with loneliness. Studies on social support suggest that daughters offer their parents more support than sons, particularly in the case of older adults, both directly and through their role as facilitators of spaces for interaction with other relatives, particularly grandchildren [62]. In addition, not all support relationships appear to be necessarily beneficial for reducing or preventing loneliness among older adults. Similar results to those of this study have been found for other outcomes, specifically mental health [31]. In this regard, support must be deployed in accordance with specific parameters depending on its source [63]. For example, previous research has shown that support from relationships (and actions) that are not perceived as relationships of support has a particularly positive impact on the psychological wellbeing of recipients. This form of invisible support [64] can be especially significant in understanding loneliness. Along these lines, sons and daughters may offer and provide effective support to their parents, but that support may arise from an interaction that older people perceive as indicative of dependence and lack of autonomy [65] to construct and maintain social relations. Similarly, the involvement of children in support relationships may indicate a lack of other social relationships and, hence, of other sources of support (such as partners, friends or siblings).

These are sound arguments. They can help to explain the pattern of association between social support from sons and daughters, on one hand, and the social and emotional dimensions of loneliness, on the other. However, they only offer a partial explanation: one might expect that the direction of the association between social support and loneliness would always be the same. In other words, these arguments do not contribute to fully explaining why support from sons and daughters is related to higher scores for social loneliness, while support from daughters is associated with lower scores for emotional loneliness. Moreover, our findings reinforce the idea that to understand the relationship between the different sources of social support and loneliness, it is necessary to develop an explanation that recognizes the complexity of this relationship as a whole, and does not merely shed light on the individual roles played by the various sources. In this regard, the qualitative material in our study offers ideas for the interpretation of our results as a whole. An analysis of this material indicates the interaction of two effects, one a cohort effect and another linked to the influence of cultural context on individual expectations regarding social relations.

The definition and characteristics of loneliness described in the introduction to this work assume that individual expectations regarding social relations are a key aspect in its development. However, these expectations arise within the framework of a specific culture, and they are hence aligned with the normative content of that culture in terms of the standards based on which relations are evaluated [66]. In this vein, as stated by Perlman [67], “loneliness is not universal; it is culture bound”, and so cultural factors affect desired levels and types of social contact, modulating its relationship with loneliness. The literature has highlighted the distinction between individualist and collectivist cultural frameworks [68]. In the latter, family relationships play a key role in the experience of loneliness among older adults. The participants in this study were born before 1960, in a context in which collectivist and family values were central elements of social life. This was also a society in which gender roles in family relationships (and specifically parent-child relationships) were firmly defined in terms of both content and relationship dynamic [69]. Specifically, for this generation the role of daughters was constructed around family, domestic and emotional elements, focusing their behaviour on caregiving [70]. This gender-based specialisation of family roles focused socialisation patterns for sons on instrumental actions, reflecting the male’s role as the family breadwinner. As can be observed, these roles imply a cultural moulding of how emotions are expressed and taken care of, a process present in the qualitative material analysed in the previous section: sons are perceived as less capable of creating a relationship of confidence, with their provision of support focused on instrumental elements, while daughters can create close bonds based on the affective dimension of social relations, which is particularly significant in reducing emotional loneliness.

We are hence confronted with a form of cohort effect, in which cultural influences play a central role. However, it is important to note that the magnitude of the association between social support from daughters and emotional loneliness is low, in addition to the fact that support from both sons and daughters is related to higher scores for social loneliness. At the same time, our results highlighted the role played by friends and siblings in understanding loneliness, particularly in its emotional dimension. We believe that as a whole, these results are due to a process of social change that may have affected cultural norms regarding social relations [71] and shaped a social context in which there is a clearly collectivist cultural pattern (in the case of the participants in our study) and another increasingly individualist cultural pattern (for the sons and daughters of the participants, who, it should be recalled, are adults and not cohabiting). The first of these patterns (collectivist) is characterised by increased connectedness of individuals to community and particularly family ties, meaning that there are high expectations in terms of the content of social interaction in the family context, particularly in the case of children. The second (individualist) produces more self-oriented and self-directed conduct, including lower expectations as to social interaction [72]. As previously noted, older people’s evaluation of social relations is influenced by normative values and expectations regarding social relations in general and intergenerational relations in particular [73]. A significant discrepancy can develop between the two above-described generational cultural patterns in this context, which might be operating to mean that older people do not find the content and nature of their relationship with their children satisfactory [see 74]. In this type of context, support received from children can even increase loneliness [66, 75], in line with the results of our research for the relationship between support from children and social loneliness. As noted by de Jong Gierveld & Tesch-Römer [71], “The alleviating effects of social integration via intergenerational family support may collapse, however, when individual living circumstances are inadequate, societal wealth marginal, and welfare state support weak. In this case, we assume that the existence of close family members and the strong normative demand to mutual support may even aggravate loneliness”.

In this context and along the lines of the convoy model [23], the results reflected in Figs 3 and 4 show the importance of support from siblings and friends in understanding loneliness [76]. Sons and daughters are not the main source of support for reducing loneliness, in fact: other relations are as significant or even more so [32]. Friendships appear to become more central to people’s lives as they age [77], particularly when family relations (above all, with spouse or children) cease to offer a source of support due to death or other reasons. The analysis of our qualitative material showed that the mechanism for constructing intimate relations is based on shared experiences. This finding reflects the proposed distinction between “significant others” (family, friends) and “similar others” (peers) coined by Thoits [78] to explain the relationship between social support and wellbeing. The former tend to form part of primary groups and have the capacity to offer general emotional support and instrumental assistance, by being physically present and making gestures that show care and concern. We can therefore identify two main mechanisms: emotional sustenance and active coping assistance. The latter (similar others) tend to be members of secondary groups and are characterised by the capacity to provide empathy and understanding in a context that requires stressor-specific emotional or instrumental support (such as shared illness or loss). Those forming part of primary groups do not generally face the same stressors and experiences of change at the same time or at the same point of the life cycle [79]. It is in these situations of change or stressors when support from similar others significantly increases in terms of its importance for psychosocial wellbeing, in our case loneliness.

However, ageing-related experiences for older adults mean that siblings and friends comprise significant others and similar others at the same time; that is, significant others with share experiences in relation to: (a) processes, changes and stressors linked to the ageing process [see 80]; and (b) changes linked to normative expectations regarding social relations, arising from the generational transition from a collectivist and family-based context (that of the cohort of study participants) to an increasingly individualist context (that of their sons and daughters) [81]. As a result, the role of siblings and friends in reducing loneliness is greatly amplified based on the construction of relationships of trust that contribute to structuring the content of daily life [82], at the same time as the role of other primary sources of support is reduced. Finally, neighbours will only play a significant part in reducing loneliness if their relationship is also one of friendship: when they become similar and significant others. Moreover, support from neighbours could arguably be perceived as a need arising from a lack of significant relationships (family and friends), which could explain the positive association between support from neighbours and the scores for emotional loneliness in the findings of this study.

### Implications for practice and future research

The present piece of research suggests implications for policies and services aimed at reducing and preventing loneliness. Our results, particularly from the qualitative phase, show that relationships of support create a sense of connectedness for older adults that directly contrasts with experiences of loneliness, largely because they develop in the natural course of interaction. In this sense, our findings reinforce other researchers’ calls to clearly focus programmes and services to better include older adults in society and maintain their social engagement [83, 84]. However, one frequent intervention practice consists of identifying at-risk groups. This identification is then used as a basis to design specific programmes aimed at reducing or preventing loneliness among those groups [85, 86], of which older adults are one example. Unfortunately, programmes designed to reduce loneliness that single out older adults may create a forced and unnatural environment for social interaction, failing to generate a sense of connection and increasing the stigma associated with loneliness [87]. Actually, our study has shown that the role of social support is best understood when considering the complexity of the (naturally occurring) social interactions in which it is generated. For example, we have found that the centrality of friendships and sibling relationships stems from their capacity to foster a sense of belonging to shared social contexts. Furthermore, the negative correlation between support from grandchildren and social loneliness suggests that these relationships contribute to the maintenance of meaningful social connections for older persons. Along these lines, we argue for the development of programmes with a population-wide approach, in line with what Dawson and Jennings [88] define as “starting with us” (and not with you or with me), reducing the emphasis on individual processes and stressing the psychosocial factors that encourage social participation and shared experiences linked to loneliness. A significant component of this social participation involves interactions with meaningful individuals from different generations. Our results indicate that relationships with younger generations, particularly children and grandchildren, play a crucial role in understanding the dynamics of loneliness among older adults. The importance of cross-generational connections may explain why intergenerational programs are particularly effective in reducing loneliness, as they foster intergenerational understanding, promote the development of high-quality social relations [89], and increase both social connectedness and cohesion [90].

Our research findings suggest lines for future research. In general, there appears to be a need for detailed descriptions outlining the content of relationships of support for the various sources, in respect of which qualitative research would be particularly useful. Some relationships of support appear especially important. Our results show that grandchildren play a highly significant role in the understanding of loneliness in general, with a particularly striking association with lower social loneliness scores. Although there is research analysing the positive effects of care from grandchildren on health and quality of life [91, 92], further studies are required to investigate the mechanisms and content of the support relationship with adolescent and adult grandchildren and its specific association with loneliness. Our results also suggest that research clarifying the statistically positive association between support from children and social loneliness would prove useful. Specifically, we would suggest a need for studies examining the potential conflict in how social relations are conceived among the cohorts of older adults and those of their sons and daughters. In this context, research analysing the role played by the reciprocity of support relationships, a factor that previous studies have linked to health and quality of life among older adults [93], would offer profound benefits in terms of knowledge regarding the determinants of loneliness. Finally, there is a need for studies addressing the role played by other potential sources of support, specifically non-family sources, resulting from changes in intergenerational patterns of care. Examples include relationships between older adults and their caregivers in developed societies, the majority of whom are immigrants [94].

### Study limitations

This study is subject to certain noteworthy limitations. First, the quantitative phase had a cross-sectional design, requiring caution when establishing causal relationships. It is also important to take into account that the relationship between social support and loneliness may be causal in one direction (levels of support influence the experience of loneliness), in the other (for example, loneliness can have an impact on relationships of support by increasing older adults’ need for support), or include both directions in a complex causal pattern. In fact, this third possibility appears the most likely. In this regard, it would be useful for future studies to use longitudinal designs that facilitate an analysis of how variations in support from multiple sources over time can influence loneliness. However, it remains the case that as our study included a qualitative phase, it made it possible to incorporate an approximate understanding of the complex relationship between social support and loneliness from the perspective of older adults themselves. Second, the scale used to evaluate loneliness (the DJGLS) is characterised by its reliability and the validity of the measures obtained. However, some authors [95] warn of the possibility of gender-based and cultural factors influencing responses to the scale. Recently, Maes et al. [96] have raised objections to some of the more commonly used scales, arguing that there is little evidence concerning test-retest reliability and measurement invariance, in addition to a significant number of the items used not appearing to be adequate indicators of loneliness. The DJGLS was not among the scales analysed, but in any event it is worth taking these observations into account. Third, this study was carried out in Spain, and caution should be exercised in any attempt to generalize the results and apply them to other countries. Both family and non-family social relations are influenced by specific cultural processes in the social context in which they take place. The same can be said of experiences of loneliness. This means that the patterns of association between both processes that our results reflect cannot be automatically applied to any other country or society.

## Conclusion

Our study shows that the relationship between social support and loneliness among older adults is notably complex, to a large degree related to the origin of the support and its different significance for the emotional and social dimensions of loneliness. In this context, the findings show that certain sources of family support may be dysfunctional and associated with increased feelings of loneliness. It appears necessary to identify the conditions in which support from family sources (particularly from children) is a protective factor, so as to innovate through intergenerational initiatives that combat loneliness. Along these same lines, non-family support is particularly effective in reducing social and emotional loneliness, provided that the social relations in question go beyond mere neighbourliness and entail the existence of links involving belonging, trust and friendship. These results point to the importance of community relationships as a context for the creation of links involving belonging. In general, the information created in this study can help to design and improve social participation, support and intervention programmes to prevent and reduce loneliness among older adults, bearing in mind that the effectiveness of social support is a key factor in maintaining an independent life, avoiding institutionalisation and reinforcing ageing in place.

## Supporting information

S1 Table Regression coefficients and covariances corresponding the structural and measurement model for emotional loneliness scores. (DOCX)

S2 Table Regression coefficients and covariances corresponding the structural and measurement model for social loneliness scores. (DOCX)

S1 File Semi-structured interview script. (PDF)
