# Supplementary material for: Unravelling the complexity of the relationship between social support sources and loneliness: A mixed-methods study with older adults
Source: PLoS One. 2025 Jan 3;20(1):e0316751. doi: 10.1371/journal.pone.0316751 (PMC11698328; doi:10.1371/journal.pone.0316751)
Supplement: S1 Table — (DOCX) [file pone.0316751.s001.docx]

**S1. Regression Coefficients and Covariances corresponding the Structural and Measurement Model for Emotional Loneliness Scores**

|  |  |  | **Estimate** | **S.E.** | **C.R.** |
| --- | --- | --- | --- | --- | --- |
| **Structural Model (latent variables)** | | |  |  |  |
| Emotional loneliness | <--- | Partner | -.074^***^ | .016 | -4.653 |
|  | <--- | Daughter(s) | -.047^*^ | .018 | -2.559 |
|  | <--- | Siblings | -.111^***^ | .020 | -5.509 |
|  | <--- | Neighbors | 5.745^***^ | 1.117 | 5.143 |
|  | <--- | Friends | -.397^***^ | .030 | -13.259 |
|  | <--- | Grandchildren | -.145^***^ | .026 | -5.602 |
| Grandchildren | <--> | Son(s) | 1.368^***^ | .129 | 10.636 |
| Grandchildren | <--> | Dauthter(s) | 1.118^***^ | .125 | 8.974 |
| Partner | <--> | Daughter(s) | 1.075^***^ | .172 | 6.268 |
| Siblings | <--> | Friends | .733^***^ | .093 | 7.893 |
| Son(s) | <--> | Daughter(s) | .624^***^ | .165 | 3.783 |
| **Measurement Model** | | |  |  |  |
| **Observed** |  | **Latent** |  |  |  |
| Emotional SS1 | <--- | Partner social support | 1.003^***^ | .007 | 144.983 |
| Emotional SS2 | <--- |  | .986^***^ | .008 | 121.333 |
| Instrumental SS1 | <--- |  | 1.000 |  |  |
| Instrumental SS2 | <--- |  | .978^***^ | .007 | 132.865 |
| Advice SS1 | <--- |  | 1.015^***^ | .006 | 167.661 |
| Advise SS2 | <--- |  | 1.011^***^ | .006 | 164.401 |
| Emotional SS1 | <--- | Siblings social support | 1.000 |  |  |
| Emotional SS2 | <--- |  | 1.027^***^ | .011 | 95.642 |
| Instrumental SS1 | <--- |  | .839^***^ | .019 | 43.890 |
| Instrumental SS2 | <--- |  | .927^***^ | .016 | 56.270 |
| Advice SS1 | <--- |  | 1.036^***^ | .012 | 89.048 |
| Advise SS2 | <--- |  | 1.041^***^ | .012 | 90.208 |
| Emotional SS1 | <--- | Neighbors social support | 1.000 |  |  |
| Emotional SS2 | <--- |  | 1.136^***^ | .135 | 8.392 |
| Instrumental SS1 | <--- |  | 1.671^***^ | .389 | 4.300 |
| Instrumental SS2 | <--- |  | 1.283^***^ | .311 | 4.132 |
| Advice SS1 | <--- |  | .896^***^ | .259 | 3.464 |
| Advise SS2 | <--- |  | 1.172^***^ | .298 | 3.931 |
| Emotional SS1 | <--- | Friends social support | 1.000 |  |  |
| Emotional SS2 | <--- |  | 1.064^***^ | .017 | 63.406 |
| Instrumental SS1 | <--- |  | 1.016^***^ | .026 | 38.555 |
| Instrumental SS2 | <--- |  | .987^***^ | .025 | 39.380 |
| Advice SS1 | <--- |  | 1.097^***^ | .021 | 52.262 |
| Advise SS2 | <--- |  | 1.114^***^ | .021 | 52.042 |
| Emotional SS1 | <--- | Son(s) social support | 1.003^***^ | .013 | 77.565 |
| Emotional SS2 | <--- |  | 1.014^***^ | .013 | 80.575 |
| Instrumental SS1 | <--- |  | .993^***^ | .009 | 107.728 |
| Instrumental SS2 | <--- |  | 1.000 |  |  |
| Advise SS1 | <--- |  | 1.037^***^ | .011 | 95.601 |
| Advice SS2 | <--- |  | 1.037^***^ | .010 | 104.756 |
| Emotional SS1 | <--- | Daughter(s) social support | 1.013^***^ | .011 | 91.172 |
| Emotional SS2 | <--- |  | 1.025^***^ | .010 | 99.789 |
| Instrumental SS1 | <--- |  | 1.005^***^ | .009 | 108.671 |
| Instrumental SS2 | <--- |  | 1.000 |  |  |
| Advise SS1 | <--- |  | 1.031^***^ | .010 | 107.450 |
| Advice SS2 | <--- |  | 1.030^***^ | .009 | 113.921 |
| Emotional SS1 | <--- | Grandchildren social support | 1.001^***^ | .020 | 50.618 |
| Emotional SS2 | <--- |  | 1.013^***^ | .018 | 56.951 |
| Instrumental SS1 | <--- |  | 1.001^***^ | .015 | 68.424 |
| Instrumental SS2 | <--- |  | 1.000 |  |  |
| Advise SS1 | <--- |  | 1.028^***^ | .016 | 63.650 |
| Advice SS2 | <--- |  | 1.039^***^ | .016 | 65.527 |
| Loneliness item 1 | <--- | Emotional loneliness | 1.000 |  |  |
| Loneliness item 4 | <--- |  | .574^***^ | .012 | 48.589 |
| Loneliness item 5 | <--- |  | .219^***^ | .008 | 28.656 |

^***^p<.001; ^**^p<.01; ^*^p<.05

Estimate: regression weights (<--) or covariances (<-->); S.E.: standard error; C.R.: critical ratio

**For every source of support (Spouse/Partner; Daughter(s); Son(s); Grandchildren; Siblings; Neighbors; Friends):**

**Emotional SS1:** Emotional social support item 1

**Emotional SS2:** Emotional social support item 2

**Instrumental SS1**: Instrumental social support item 1

**Instrumental SS2**: Instrumental social support item 2

**Advise SS1**: Advise social support item 1

**Advice SS2**: Advise social support item 2
