# Supplementary material for: Unravelling the complexity of the relationship between social support sources and loneliness: A mixed-methods study with older adults
Source: PLoS One. 2025 Jan 3;20(1):e0316751. doi: 10.1371/journal.pone.0316751 (PMC11698328; doi:10.1371/journal.pone.0316751)
Supplement: S2 Table — (DOCX) [file pone.0316751.s002.docx]

**S2. Regression Coefficients and Covariances corresponding the Structural and Measurement Model for Social Loneliness Scores**

|  |  |  | **Estimate** | **S.E.** | **C.R.** |
| --- | --- | --- | --- | --- | --- |
| **Structural Model (latent variables)** | | |  |  |  |
| Social loneliness | <--- | Partner | -.293^***^ | .039 | -7.422 |
|  | <--- | Son(s) | .753^***^ | .104 | 7.256 |
|  | <--- | Daughter(s) | .940^***^ | .124 | 7.579 |
|  | <--- | Siblings | -.058^**^ | .022 | -2.681 |
|  | <--- | Friends | -.265^***^ | .032 | -8.193 |
|  | <--- | Grandchildren | -3.773^***^ | .422 | -8.943 |
| Grandchildren | <--> | Daughter(s) | 1.481^***^ | .128 | 11.573 |
| Grandchildren | <--> | Son(s) | 1.212^***^ | .119 | 10.183 |
| Daughter(s) | <--> | Partner | 1.137^***^ | .163 | 6.966 |
| Daughter(s) | <--> | Son(s) | .642^***^ | .166 | 3.867 |
| Neighbors | <--> | Friends | .397^***^ | .045 | 8.725 |
| Siblings | <--> | Friends | .531^***^ | .083 | 6.390 |
| **Measurement Model** | | |  |  |  |
| **Observed** |  | **Latent** |  |  |  |
| Emotional SS1 | <--- | Partner social support | .986^***^ | .008 | 121.583 |
| Emotional SS2 | <--- |  | 1.003^***^ | .007 | 145.284 |
| Instrumental SS1 | <--- |  | 1.000 |  |  |
| Instrumental SS2 | <--- |  | .978^***^ | .007 | 133.161 |
| Advice SS1 | <--- |  | 1.011^***^ | .006 | 164.757 |
| Advice SS2 | <--- |  | 1.015^***^ | .006 | 168.011 |
| Emotional SS1 | <--- | Siblings social support | 1.000 |  |  |
| Emotional SS2 | <--- |  | 1.027^***^ | .011 | 94.722 |
| Instrumental SS1 | <--- |  | .839^***^ | .019 | 43.459 |
| Instrumental SS2 | <--- |  | .927^***^ | .017 | 55.715 |
| Advice SS1 | <--- |  | 1.036^***^ | .012 | 88.170 |
| Advice SS2 | <--- |  | 1.041^***^ | .012 | 89.360 |
| Emotional SS1 | <--- | Friends social support | 1.000 |  |  |
| Emotional SS2 | <--- |  | 1.064^***^ | .017 | 61.300 |
| Instrumental SS1 | <--- |  | 1.016^***^ | .027 | 37.225 |
| Instrumental SS2 | <--- |  | .987^***^ | .026 | 38.007 |
| Advice SS1 | <--- |  | 1.097^***^ | .022 | 50.459 |
| Advice SS2 | <--- |  | 1.115^***^ | .022 | 50.295 |
| Emotional SS1 | <--- | Son(s) social support | 1.003^***^ | .013 | 77.745 |
| Emotional SS2 | <--- |  | 1.014^***^ | .013 | 80.770 |
| Instrumental SS1 | <--- |  | .993^***^ | .009 | 107.981 |
| Instrumental SS2 | <--- |  | 1.000 |  |  |
| Advise SS1 | <--- |  | 1.037^***^ | .011 | 95.815 |
| Advise SS2 | <--- |  | 1.037^***^ | .010 | 104.985 |
| Emotional SS1 | <--- | Daughter(s) social support | 1.013^***^ | .011 | 91.653 |
| Emotional SS2 | <--- |  | 1.025^***^ | .010 | 100.323 |
| Instrumental SS1 | <--- |  | 1.005^***^ | .009 | 109.224 |
| Instrumental SS2 | <--- |  | 1.000 |  |  |
| Advise SS1 | <--- |  | 1.031^***^ | .010 | 108.009 |
| Advise SS2 | <--- |  | 1.030^***^ | .009 | 114.494 |
| Emotional SS1 | <--- | Grandchildren social support | 1.002^***^ | .080 | 12.588 |
| Emotional SS2 | <--- |  | .995^***^ | .078 | 12.692 |
| Instrumental SS1 | <--- |  | .981^***^ | .026 | 38.350 |
| Instrumental SS2 | <--- |  | 1.000 |  |  |
| Advise SS1 | <--- |  | .963^***^ | .077 | 12.500 |
| Advise SS2 | <--- |  | .972^***^ | .027 | 36.408 |
| Emotional SS1 | <--- | Neighbors social support | 1.000 |  |  |
| Emotional SS2 | <--- |  | 1.160^***^ | .031 | 37.691 |
| Instrumental SS1 | <--- |  | 1.059^***^ | .043 | 24.589 |
| Instrumental SS2 | <--- |  | .924^***^ | .037 | 24.957 |
| Advice SS1 | <--- |  | 1.174^***^ | .035 | 33.721 |
| Advice SS2 | <--- |  | 1.199^***^ | .035 | 34.127 |
| Loneliness item 2 | <--- | Social loneliness | 1.000 |  |  |
| Loneliness item 3 | <--- |  | .364*** | .009 | 38.451 |
| Loneliness item 6 | <--- |  | .529*** | .010 | 51.389 |

^***^p<.001; ^**^p<.01; ^*^p<.05

Estimate: regression weights (<--) or covariances (<-->); S.E.: standard error; C.R.: critical ratio

**For every source of support (Spouse/Partner; Daughter(s); Son(s); Grandchildren; Siblings; Neighbors; Friends):**

**Emotional SS1:** Emotional social support item 1

**Emotional SS2:** Emotional social support item 2

**Instrumental SS1**: Instrumental social support item 1

**Instrumental SS2**: Instrumental social support item 2

**Advise SS1**: Advise social support item 1

**Advice SS2**: Advise social support item 2
