# Supplementary material for: Unravelling the complexity of the relationship between social support sources and loneliness: A mixed-methods study with older adults
Source: PLoS One. 2025 Jan 3;20(1):e0316751. doi: 10.1371/journal.pone.0316751 (PMC11698328; doi:10.1371/journal.pone.0316751)
Supplement: S1 File — (PDF) [file pone.0316751.s003.pdf]

Semi-Structured Interview Script

Introduction / Ice breakers

Let's talk about the people you interact with on a daily basis, as well as the people you turn to when you have a problem, feel lonely, or even when you want to talk about your life.

- On a typical day, who do you interact with? Of those people, who are the most important to you?
- If you need something, who do you turn to? For example, if you get sick, if you need a ride somewhere, or if you have financial problems.
- Do you meet these people often, or would you like to see them more frequently? Is there anyone in particular you would like to meet more?
- Is there anyone who, unexpectedly, has become indispensable in your life recently?
- As a [man/woman], do you think the relationships you have at this stage of your life with the people around you are different from those of a [woman/man]? How are they different?
- In what situations do you most value receiving help or being able to turn to someone?
  REMINDER TO INTERVIEWER: Only ask if NOT mentioned: When do you feel lonely, when do you need help, when do you have financial needs?
- What kind of support is most important to you?
  REMINDER TO INTERVIEWER: If unsure, provide examples: someone to listen to you, give you advice, or talk to about your feelings; or someone to support you with daily tasks, like taking care of your home, accompanying you to the doctor, etc.

For <u>nursing home</u> residents only:

- Are you satisfied with your relationships with other people living in the residence?
- What do your relationships with the people living in the residence provide you?
- How did you expect your relationships to be before moving into the residence?
- What change would you expect in your relationships with others to be more satisfied?

For all participants:

- Do you feel lonely? Would you say you are lonely?
  REMINDER TO INTERVIEWER: We aim to distinguish between emotional loneliness and social isolation. For example: Do you feel lonely, or do you feel you have no one to share your experiences with? Do not suggest an answer.
- In what situations do you feel most lonely?
- In what situations do you feel most accompanied, that you are not alone?
- Do you feel that at this stage of life it is more likely/normal/frequent to feel lonely? Why?
- From your perspective, what circumstances contribute to feeling lonely as we age? **REMINDER TO INTERVIEWER:** We are trying to identify the reasons for feeling lonely.
